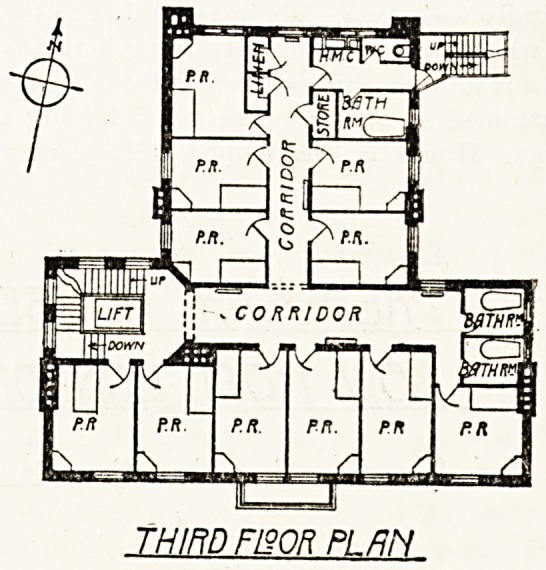# Tredegar House, Bow

**Published:** 1912-07-20

**Authors:** 


### TREDEGAR HOUSE, BOW.

### The London Hospital's New Preliminary Training School.

There are in the Metropolis many less attractive thoroughfares, so far as buildings are concerned, than Bow Road, E.; and Mr. Rowland Plumbe, F.R.I.B.A., has made the elevation of new Tredegar House most attractive and inviting architecturally. The interior of the building is attractive, too, and everything, though simple, is exceedingly well done. We imagine that to the majority of those who seek preliminary training here the building will offer advantages and comforts greater than they have been accustomed to before. The great feature and lesson of Tredegar House is the emphasis it places upon the importance of providing that candidates desirous of becoming regular probationers in a great hospital should reside outside and not form a part of the ordinary establishment of the institution, until the preliminary period during which their suitability to become qualified probationers has been successfully passed.

### An Important Principle.

It is an important principle here that every pupil-probationer shall occupy a separate bedroom, and enjoy the advantages of comfortable domestic arrangements, excellent bath-rooms, class-rooms and garden, and also a playground on the roof, from which there are extended views. Tredegar House was opened by Queen Alexandra, the President of the London Hospital, on July 19, and we feel confident her Majesty must have been deeply interested in the system of training, and especially in the arrangements made for teaching sick-room cookery, the art of bandaging and many details of practical nursing.

### The Staff Accommodation.

We were glad to notice that importance has been wisely given to the provision of most attractive accommodation for the sister-in-charge and her two assistants. It is no easy task to discharge successfully the duties of sister-in-charge, who must be a lady of not only considerable nursing experience but a woman who is a disciplinarian and who possesses character as well as sound knowledge. Upon her depends largely the success of the preliminary training school, for there in the course of six weeks' residence the sister has to select and report which of the candidates are suitable to become probationer-nurses and which are not.

The justification of the extra expense involved in attaching a preliminary sifting-house to each large nurse-training school in the present day reveals at once the importance of keeping the novices separated from the hospital nursing staff.

We are indebted to Mr. Rowland Plumbe, the architect, for the plans of Tredegar House, which we have the pleasure to publish this week. The London Hospital is indebted to Lord Tredegar for his generous gift of the site of Tredegar House and to those members of the public who have contributed to the cost of the new buildings, in testimony of their gratitude for the aid rendered to themselves and their families through the services of the private nursing staff.

THE GROUND FLOOR AND HALL.

The house is entered by a flight of steps. On the ground floor there is an ample entrance-hall, a sitting-room for the sister-in-charge, visitors' room, and probationers' sitting-room. These all face the road. Crossing the hall, which contains a lift, we enter the lobby, having on the right a servery, on the left a spacious lavatory, and beyond these open out the probationers' dining-room and a large lecture-room both communicating with and looking out on to the garden.

On the first floor are two small class-rooms, and, with the second, third, and fourth floors, provide accommodation for probationers, sisters, and servants, with the necessary offices, linen stores, etc., as shown on the accompanying plans. The total accommodation consists of separate bedrooms for thirty pupils, with three rooms for sisters and three for the servants. A spare room is also provided on the first floor. The house is well lighted, attractive, and comfortable. There is an electric lift com-

FIRST FLOOR PLAN

municating with all floors in addition to the main staircase.

THE KITCHENS AND BASEMENT.

The basement, which is really the lower ground floor—seeing that the buildings are entirely detached all round with good wide areas, and that the basement floor is arranged only six feet below the level of the garden and front forecourt—forms an important feature of the building. Here will be found a large kitchen with working pantry and scullery, having a lift to the dining-room, also larders and store-rooms, the latter including a blanket and linen store and a mending-room, and also a box-room. Here, too, is a servants' hall, with a bathroom and lavatory, a porters' room, the heating chamber, and cold store. The tradesmen's entrance is situated in the front area, so that the entrance hall can be used wholly for the purpose of the training school.

THE SANITATION.

The architect, both on account of space, economy, and customary usage, has not provided sanitary spurs. He points out that the building is in no sense a hospital, being provided for probationers who are going through their preliminary training before going to the hospital. The facilities the plan affords for the work of the training

THIRD FLOOR PLAN

school and for the comfort of its inmates will no doubt make it a great and popular addition to the teaching forces of the London Hospital. Tredegar House should impress every woman who passes through it with a sense of the thoroughness of the training and the due importance with which she should regard her acceptance as a probationer at this large and successful training school for nurses.

## Figures and Tables

**Figure f1:**
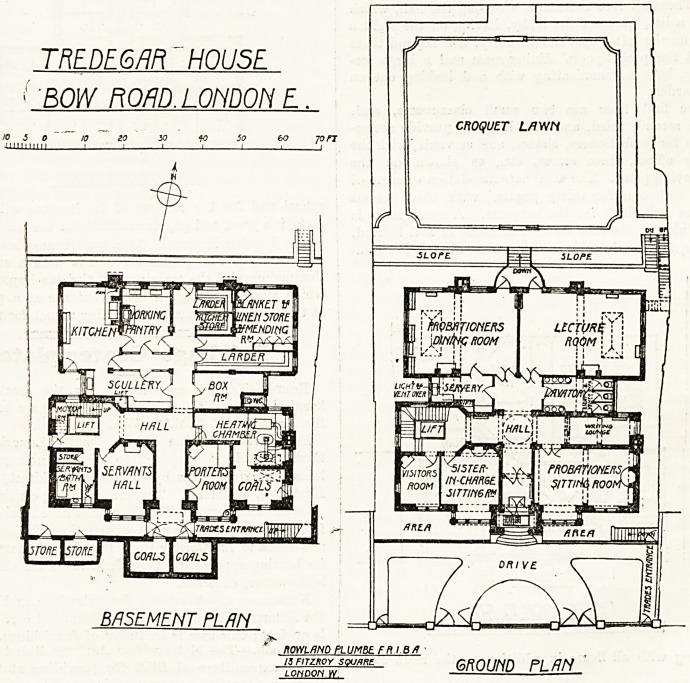


**Figure f2:**
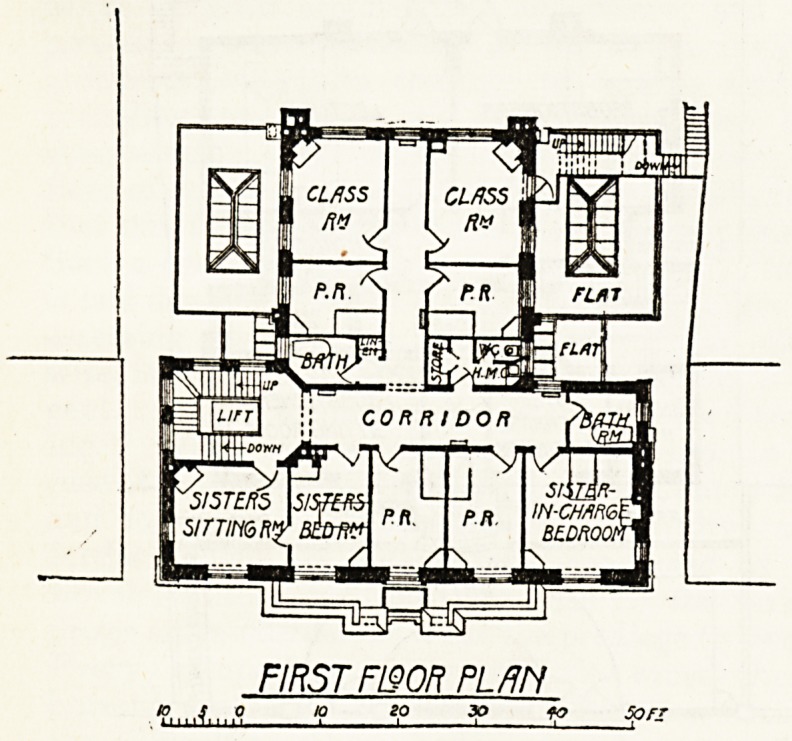


**Figure f3:**